# Characteristics and Outcomes of Pediatric COVID-19 Patients in Osaka, Japan

**DOI:** 10.3390/ijerph18115911

**Published:** 2021-05-31

**Authors:** Yusuke Katayama, Ling Zha, Tetsuhisa Kitamura, Atsushi Hirayama, Taro Takeuchi, Kenta Tanaka, Sho Komukai, Takeshi Shimazu, Tomotaka Sobue, on behalf of the COVID-19 Epidemiology Research Group of Osaka University

**Affiliations:** 1 Department of Traumatology and Acute Critical Medicine, Graduate School of Medicine, Osaka University, Suita, Osaka 565-0871, Japan; orion13@hp-emerg.med.osaka-u.ac.jp (Y.K.); shimazu@hp-emerg.med.osaka-u.ac.jp (T.S.); 2 Department of Social and Environmental Medicine, Division of Environmental Medicine and Population Sciences, Graduate School of Medicine, Osaka University, Suita 565-0871, Japan; ivy_mist@outlook.com (L.Z.); tarogauss106072@gmail.com (T.T.); tanaken.0414@gmail.com (K.T.); tsobue@envi.med.osaka-u.ac.jp (T.S.); 3 Department of Social Medicine, Division of Public Health, Graduate School of Medicine, Osaka University, Suita, Osaka 565-0871, Japan; atsushihirayamamd@gmail.com; 4 Department of Integrated Medicine, Division of Biomedical Statistics, Graduate School of Medicine, Osaka University, Suita, Osaka 565-0871, Japan; skomukai@biostat.med.osaka-u.ac.jp

**Keywords:** SARS-CoV-2, epidemiology, children, adolescents, Japan

## Abstract

The epidemiological information on characteristics, in-hospital treatments, and outcomes of the coronavirus disease 2019 (COVID-19) among pediatric patients has not been fully evaluated in Japan. This was a retrospective observational study conducted in the Osaka Prefecture, Japan, and we enrolled laboratory-confirmed COVID-19 patients aged ≤19 years old from January to November in 2020. Of 14,846 COVID-19 eligible patients, 1240 pediatric patients (8.4%) were registered during the study period; 329 were children aged 0–9 years (26.5%) and 911 were adolescents aged 10–19 years (73.5%). The majority of the patients exhibited mild symptoms at diagnosis (872, 70.3%), some were asymptomatic (296, 23.9%). Cluster infections occurred in child-care facilities (26, 7.9%) among children and in universities (27, 3.0%) and schools (18, 2.0%) among adolescents. The number of close-contact cases was 260 (69.0%) in children and 459 (50.4%) in adolescents. Sixty of the children (18.2%) and 90 of the adolescents (9.9%) were hospitalized. One patient received mechanical ventilation, and none underwent extracorporeal membrane oxygenation. One patient was admitted to the intensive care unit; there were no deaths. These results are useful for recognizing the clinical course from transmission route to outcomes of this infection in pediatric patients.

## 1. Introduction

The coronavirus disease 2019 (COVID-19), which was identified in Wuhan, China, in December 2019, has spread globally [1]. Pediatric patients accounted for only 1–2% of all those infected with COVID-19 worldwide [2,3]. Unlike in the case of other respiratory infectious diseases, children may have a lower incidence risk of COVID-19 than adults. Most pediatric patients with COVID-19 exhibit mild symptoms or are asymptomatic, and only a few fatal cases have been reported [4,5,6]. In fact, the proportion of pediatric patients with severe COVID-19 was only 0.6%, as published in the first report from China [4].

Previous studies from China, the United States, and European countries reported that the common symptoms of COVID-19 among pediatric patients were fever, cough, and nausea/vomiting [4], although most of them were asymptomatic [4,7,8]. Additionally, many pediatric patients were infected in their homes [8,9,10]. While some adults with COVID-19 develop severe respiratory failure, some children develop symptoms of Kawasaki disease and toxic syndrome [11,12,13], which was later characterized as multisystem hyperinflammatory syndrome in children (MIS-C); this may prove to be a significant problem in Japan in the future. Further, the epidemiological information on characteristics, in-hospital treatments, and outcomes of COVID-19 among pediatric patients has not been fully evaluated in Japan.

In Japan, the first case of COVID-19 was identified on 15 January 2020, and a total of 100,762 cases had been confirmed by the end of October 2020 [14]. The Osaka Prefecture is the largest metropolitan area in Western Japan, and after Tokyo, it has the second highest number of COVID-19 cases. The aim of this study was to determine the characteristics and outcomes of pediatric COVID-19 patients aged 0–19 years in the Osaka Prefecture, Japan, using the COVID-19 patient registry managed by the Osaka Prefectural Government.

## 2. Materials and Methods

### 2.1. Population, Design, and Setting

This retrospective observation was conducted in the Osaka Prefecture, Japan, between January 2020 and November 2020. Based on the Infectious Diseases Control Law, an active investigation was conducted to collect the epidemiological information on COVID-19 patients by using a data-collection system with a uniform format managed by the Osaka Prefectural Government [15]. Until October 2020, all COVID-19 cases were registered on this system. In November 2020, this system and the government-recommended system were used together, and it has been fully integrated into the government-recommended system (health center real-time information-sharing system on COVID-19: HER-SYS) [16] since December 2020. Therefore, not all the cases in November 2020 were registered.

The Osaka Prefecture is located in the central area of Western Japan and covers an area of 1905 km^2^. The estimated population was 8,819,226, as of April 1, 2020 [17]. Local public health centers collected data of all the patients who tested positive for COVID-19 through telephone or electronic worksheets. All COVID-19 cases that were diagnosed at hospitals or testing centers through laboratory tests were reported to the local health center. The coordinators in admission follow-up centers or public health centers decided the patient’s care course (hospitalization, recuperation at accommodation facilities, and recuperation at home) based on his or her vital signs, symptoms, age, and comorbidities. The collected data were reported to the Osaka Prefectural Government and the Ministry of Health, Labor and Welfare [18]. The release criteria from isolation for symptomatic patients with COVID-19 was as follows: 10 days passed after symptom onset, and at least 3 days passed without any symptoms, including fever and/or respiratory-related symptoms, or two consecutive negative results such as polymerase chain reaction (PCR) method at least 24-h apart. In addition, the follow-up release criterion for asymptomatic patients was 10 days after a positive test for COVID-19 or two consecutive negative results at least 24-h apart 6 days after a positive test [19]. Among the COVID-19 patients registered on the data collection system between January 2020 and November 2020, we obtained eligible cases with complete follow-up from the Osaka Prefecture.

### 2.2. Measurements

The following information associated with the COVID-19 cases from Osaka Prefecture were collected: age; sex; underlying medical conditions; residence; cluster; close contacts; symptoms at diagnosis; hospitalization; death; date of symptom onset; date of hospitalization; date of death (for patients who died during the observation period); treatments, such as oxygen therapy, mechanical ventilation, intensive care unit (ICU) admission, renal replacement therapy, and extracorporeal membrane oxygenation (ECMO). Age groups were categorized as units of 10 years in the provided data, because the Osaka Prefecture did not provide detailed age as publicly available data to protect personal information among patients with COVID-19. Therefore, we defined children as those aged 0–9 years and adolescents as those aged 10–19 years.

Further, the time period between 1 February 2020 and 13 June 2020 was considered as the first surge, that of the second surge being between 14 June 2020 and 9 October 2020, and the rise in cases after 10 October 2020 was considered the third surge [19]. Patients with underlying medical conditions, such as diabetes; heart failure; respiratory diseases, including chronic obstructive pulmonary disease, chronic kidney diseases requiring dialysis; and the use of immunosuppressants and anticancer drugs were considered high risk, and this information was summarized in the database [19]. Cluster was defined as a group of ≥5 patients positive with COVID-19 who had an epidemiological link with the index patient identified in establishments, such as medical institutions, universities, and child-care facilities [20]. Close contacts were identified by local public health centers as those who lived with an infected patient or had prolonged contact with the patient, those who examined, nursed, or cared for the patient without protection, those who were likely to have had direct contact with contaminated materials, or those who had contact with the patient for more than 15 min at short distance (approximately 1 m) without protection [20]. Symptoms at diagnosis were defined as follows: mild (only cough without breathlessness or respiratory symptoms), moderate (breathlessness, pneumonia, or the need for oxygen therapy), and severe (admission to the ICU or the use of mechanical ventilation) [16]. Clusters in this study were categorized into the following groups: no, medical institution, university, school, child facility, and others. The onset date was defined as the date when any symptoms were estimated to have appeared [21,22]. If the onset date was missing, we substituted it with the date of medical treatment, the date of hospital admission, or the date when a change in symptoms occurred, whichever came first. For the hospitalized patients, the following data were included: date of hospitalization and/or discharge, reason for discharge (alive or death).

### 2.3. Statistical Analysis

Categorical variables are presented as numbers and percentages. Continuous variables were presented as median and interquartile range (IQR). The chi-square test or Fisher’s exact test was used to compare categorical variables, and the Wilcoxon rank-sum test was used to compare continuous variables between the age groups. The weekly distribution of the number of COVID-19 patients by age group is described considering 1 January 2020 as the first day of the week. All tests were two-tailed, and a *p* value <0.05 was considered statistically significant. Statistical analyses were performed using Stata version 14 (StataCorp. 2015, College Station, TX, USA).

## 3. Results

### 3.1. Eligible Patients

**Figure 1:**
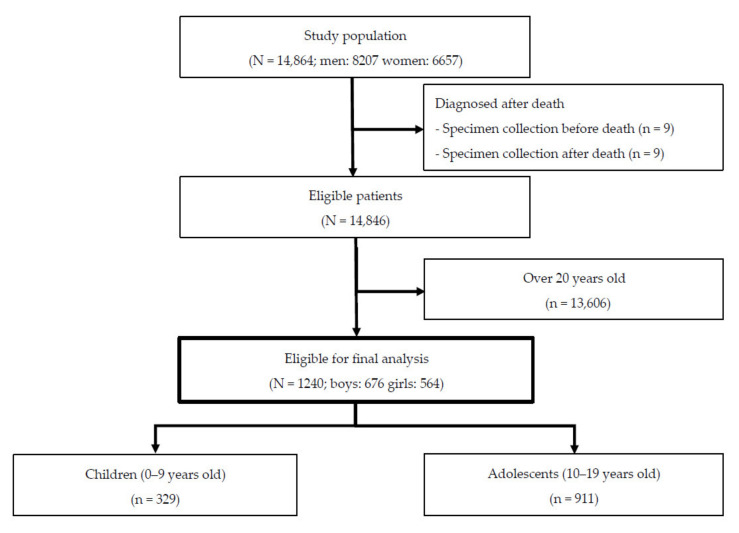
Patient flowchart.

Of the 14,846 eligible patients with COVID-19, 1240 pediatric patients (8.4%) were registered during the study period, of which 329 were children and 911 were adolescents (Figure 1).

### 3.2. The Description of Baseline Features

Table 1 shows the clinical and demographic characteristics of the pediatric patients with COVID-19. There were 164 boys (49.8%) among the children, while there were 512 boys (56.2%) among the adolescents (*p* = 0.047). Most of the children lived in Osaka City (177, 53.8%); however, most of the adolescents (541, 59.4%) lived in other areas of the Osaka Prefecture (*p* < 0.001). Sixty pediatric patients had underlying medical conditions. Majority of the cases were mild at the time of diagnosis (872 cases, 70.3%), while some were asymptomatic (296 cases, 23.9%). There were 79 patients (6.4%) in the first surge of COVID-19, 838 (67.6%) in the second surge, and 323 (26.0%) in the third surge. Clusters occurred in child-care facilities (26, 7.9%) among children, and in universities (27, 3.0%) and schools (18, 2.0%) among adolescents. The number of close contact cases was 260 (69.0%) among the children and 459 (50.4%) among the adolescents (*p* < 0.001). Sixty of the children (18.2%) were hospitalized, while among the adolescents, 90 (9.9%) were hospitalized (*p* < 0.001).

### 3.3. Weekly Incident Pattern of Children with COVID-19

**Figure 2:**
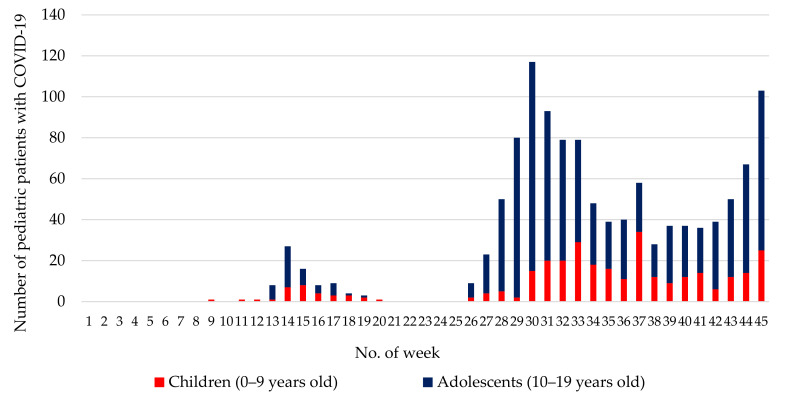
Weekly number of pediatric COVID-19 patients. A data collection system managed by Osaka Prefecture was used until October, 2020. This system was used with the government-recommended one in November 2020 and has been fully integrated into the government-recommended system since December 2020; therefore, not all cases in November 2020 were registered.

Figure 2 shows the weekly number of pediatric patients with COVID-19. Among the children, the highest number was in week 37 (34 cases; 3–9 September), while among the adolescents, week 30 (102 cases; 16–22 July) had the highest number of cases.

### 3.4. The Description of Clinical Features

Table 2 shows the clinical characteristics of the hospitalized pediatric patients. The median number of days from onset to hospitalization was 4 days (IQR, 2–7 days). The median length of hospital stay was 8 days (IQR, 6–11 days) in total; 8.5 days (IQR, 6–12 days) in children and 8 days (IQR, 6–10 days) in adolescents (*p* = 0.234). One patient received mechanical ventilation, and none of them received ECMO. One patient was admitted to the ICU; there were no deaths.

## 4. Discussion

This was the first study to reveal the detailed characteristics and outcomes of pediatric patients with COVID-19 aged 0–19 years in Osaka Prefecture, Japan. The proportion of children and adolescents with COVID-19 was <10%. Most pediatric patients exhibited mild symptoms, and the proportion of asymptomatic patients was about one-fourth of the total. Only a few of the patients were hospitalized, one patient was admitted to the ICU, and no deaths were observed. We believe that this study would help us understand the actual situation of COVID-19 infection among pediatric patients and prevent its transmission.

In this study, the proportion of asymptomatic pediatric patients at diagnosis was about one-fourth of the total; however, in previous studies, the proportion of asymptomatic patients was higher in children and adolescents than in adults [2,23]. In an international study of pediatric COVID-19 patients aged <18 years in European countries, 16% of them were asymptomatic at the time of visit to hospitals and clinics [13]. In China, 12.9–15.8% of COVID-19 pediatric patients were asymptomatic [4,6]. Thus, the proportion of asymptomatic pediatric patients seemed to be higher in Japan than in other countries. The reason for this difference is unclear. In Japan, since the first case of COVID-19 was reported, public health officers have been conducting an active investigation, including PCR testing of relevant persons who had been in contact with COVID-19 patients such as family members and friends. During the study period, the number of COVID-19 patients in Japan was considerably lower than in the United States and European countries [24]. Therefore, active follow-up of clusters and close contacts may be associated with a higher proportion of asymptomatic patients. Nevertheless, the identification of infected patients and the tracing of close contacts by public health officers are crucial to prevent the spread of COVID-19.

Considering the transmission route, ≥90% of pediatric COVID-19 cases did not occur in clusters. Among children, 7.9% of cluster cases occurred in child-care facilities, and among adolescents, 5.0% of cluster cases were seen in schools and universities. The proportion of COVID-19 infection from close contacts was higher in children than in adolescents. Generally, children were reported to be infected with COVID-19 in their homes [9,10,25], with a previous study from China showing that 90% of pediatric COVID-19 patients were infected by family members [6] and another study from Australia demonstrating a low rate of COVID-19 transmission in educational settings [26]. In northern Italy, the proportion of COVID-19 infection in schools was low, although there was an incidence of a cluster with ≥10 patients in a high school [27,28]. Teenagers engaged in a wide range of activities may be at a higher risk of infection outside their homes, such as from attending schools or universities, whereas infants and toddlers may have higher chance of being exposed to infection inside their homes. Parents or other family members who get infected with COVID-19 from outside their homes could transmit it to their children and adolescents at home. However, many infected children and adolescents are asymptomatic and can spread the infection to their family members, some of whom develop severe symptoms [23]. Therefore, it is important that all family members take thorough precautions to prevent COVID-19 transmission, even in homes, by wearing masks and disinfecting hands with alcohol.

The proportion of cluster infections in child-care facilities was high among children, which may be because toddlers were more likely to engage in high-risk behaviors, such as licking and putting things in their mouths. Interestingly, COVID-19 infections among children decreased in the Osaka Prefecture during weeks 32–35 in July and August when public elementary schools and kindergartens were closed for the summer vacation. However, in week 37, two weeks after the reopening of public elementary schools and kindergartens, there was a rise in the number of infections among children. Therefore, thorough infection control measures are essential, especially for children attending elementary schools and kindergartens.

In this study, no deaths were observed among the pediatric patients. Several previous studies have shown that the mortality rate of pediatric COVID-19 patients was <2% [12,13,29,30,31], and the outcomes were better in children and adolescents than in adults and older individuals. Further, only one patient was admitted to the ICU in this study, whereas the proportion of ICU admissions reported in other studies worldwide varied from 8.0% to 73.8% [12,13,29,30]. Thus, there could be racial and regional differences in the severity and mortality rates among pediatric COVID-19 patients; investigating this would require further observational studies.

This study has several limitations. First, the patients were grouped according to age in categories of 10 years each, because the Osaka Prefecture did not provide detailed age as publicly available data to protect personal information among patients with COVID-19. Therefore, we were unable to analyze the data using detailed age categories such as infant aged <1 year, toddler to preschooler aged 1–5 years, school-aged child aged 6–13 years, and adolescent aged 14–19 years, because the characteristics and outcomes of pediatric COVID-19 patients might be affected by the level of physical development, hormonal status, and educational institution at each developmental stage. Thus, further understanding the features of Japanese COVID-19 patients using detailed age is an important issue to be addressed in the future. Second, we analyzed the epidemiological data collected by the public health centers under the Infectious Diseases Control Law; therefore, clinical data such as blood tests and medications could not be assessed. Third, underlying medical conditions, such as hypertension and diabetes mellitus, were aggregated in this registry, and there was no information on food allergy or asthma. Therefore, we were not able to determine the relationship between pediatric patients with COVID-19 and the underlying medical conditions. Fourth, the data provided by the Osaka Prefecture did not include all cases (e.g., incomplete follow-up cases were excluded from the provided data). Therefore, the number of cases, treatments, and other information available on the website of Osaka Prefecture were different from our results. Finally, we did not obtain information on MIS-C, which is of interest in pediatric COVID-19 patients. Despite these limitations, our study findings are important as they provide fundamental learning regarding the epidemiology of pediatric COVID-19 patients in Japan.

## 5. Conclusions

Using the comprehensive registry covering the entire Osaka Prefecture, Japan, we revealed the clinical course from transmission route to outcomes of pediatric patients infected with COVID-19. Pediatric patients with COVID-19 accounted for 8.4% of the total cases, and no deaths were observed. Most of the study subjects exhibited mild symptoms, while a few were asymptomatic. Among children, cluster infections frequently occurred in child care facilities, and among adolescents, clusters were seen in schools and universities. Therefore, thorough precautions to prevent COVID-19 transmission are essential, especially for children attending educational institutions as transmission route.

## Figures and Tables

**Table 1:** Epidemiological characteristics of pediatric patients in Osaka Prefecture, Japan.

Factor	Total		Children		Adolescents		*p*-Value
			(0–9 Years Old)		(10–19 Years Old)		
	*N*	(%)	*N*	(%)	*N*	(%)	
No. of patients	1240		329		911		
Sex							
Boys	676	(54.5)	164	(49.8)	512	(56.2)	0.047 ^a^
Girls	564	(45.5)	165	(50.2)	399	(43.8)	
Geographic area							
Osaka City	538	(43.4)	177	(53.8)	361	(39.6)	<0.001 ^b^
Other areas in Osaka	692	(55.8)	151	(45.9)	541	(59.4)	
Outside Osaka Prefecture	8	(0.6)	1	(0.3)	7	(0.8)	
Unknown	2	(0.2)	0	(0.0)	2	(0.2)	
Underlying medical conditions							
No	1180	(95.2)	312	(94.8)	868	(95.3)	0.746 ^a^
Yes	60	(4.8)	17	(5.2)	43	(4.7)	
Disease severity at diagnosis							
Asymptomatic	296	(23.9)	124	(37.7)	172	(18.9)	<0.001 ^b^
Mild	872	(70.3)	175	(53.2)	697	(76.5)	
Moderate	1	(0.1)	1	(0.3)	0	(0.0)	
Severe	1	(0.1)	0	(0.0)	1	(0.1)	
Unknown	70	(5.6)	29	(8.8)	41	(4.5)	
Surge							
First (~13 Jun.)	79	(6.4)	32	(9.7)	47	(5.2)	0.012 ^a^
Second (14 Jun ~9 Oct)	838	(67.6)	219	(66.6)	619	(67.9)	
Third (10 Oct ~)	323	(26.0)	78	(23.7)	245	(26.9)	
Cluster							
No	1152	(92.9)	297	(90.3)	855	(93.9)	<0.001 ^b^
Child facility	28	(2.3)	26	(7.9)	2	(0.2)	
University	27	(2.2)	0	(0.0)	27	(3.0)	
School	20	(1.6)	2	(0.6)	18	(2.0)	
Medical institution	7	(0.6)	4	(1.2)	3	(0.3)	
Other clusters	6	(0.5)	0	(0.0)	6	(0.7)	
Close contact							
No	521	(42.0)	69	(21.0)	452	(49.6)	<0.001 ^a^
Yes	719	(58.0)	260	(79.0)	459	(50.4)	
Hospitalization							
No	1082	(87.3)	268	(81.5)	814	(89.4)	<0.001 ^a^
Yes	150	(12.1)	60	(18.2)	90	(9.9)	
Unknown	8	(0.6)	1	(0.3)	7	(0.8)	

^a^ Groups compared using Pearson’ s chi-squared. ^b^ Groups compared using Fisher’ s exact test.

**Table 2:** Clinical characteristics of hospitalized pediatric patients in Osaka Prefecture, Japan.

Factor	Total		0–9 Years Old		10–19 Years Old		*p*-Value
No. of hospitalized patients	150		60		90		
Days to hospitalization, median (IQR)	4	(2, 7)	3	(2, 6)	4	(2, 7)	0.437 ^a^
Length of hospital stay, median (IQR)	8	(6, 11)	8.5	(6, 12)	8	(6, 10)	0.234 ^a^
Oxygen therapy, *n* (%)	2	(1.3)	1	(1.7)	1	(1.1)	1.000 ^b^
Mechanical ventilator, *n* (%)	1	(0.7)	0	(0.0)	1	(1.1)	1.000 ^b^
Intensive care unit, *n* (%)	1	(0.7)	0	(0.0)	1	(1.1)	1.000 ^b^
Renal replacement therapy, *n* (%)	0	(0.0)	0	(0.0)	0	(0.0)	NA
Extracorporeal membrane oxygenation, *n* (%)	0	(0.0)	0	(0.0)	0	(0.0)	NA
Death, *n* (%)	0	(0.0)	0	(0.0)	0	(0.0)	NA

IQR, interquartile range; NA, not available. ^a^ Groups compared using Wilcoxon rank-sum test. ^b^ Groups compared using Fisher’s exact test.

## Data Availability

Data sharing not applicable.
